# Hydatid cyst of the calf presenting as painless mass: A case report

**DOI:** 10.1016/j.ijscr.2019.06.042

**Published:** 2019-06-26

**Authors:** Ayad Ahmad Mohammed, Sardar Hassan Arif

**Affiliations:** University of Duhok, College of Medicine, Department of Surgery, Iraq

**Keywords:** Hydatid disease, *Echinococcus granulosus*, Echinococcosis, Tape worm, Musculoskeletal system, Case report

## Abstract

**Background:**

Hydatid disease is a zoonotic disease that is transmitted to the human by ingesting the eggs of the parasite *Echinococcus granulosus*, it most commonly affects the liver but every organ could be affected, musculoskeletal system involvement occurs in 0.5–4% of patients. Most patients present as soft tissue mass in the affected muscle.

Preoperative diagnosis usually done radiologically by ultrasound or MRI examination.

**Case presentation:**

We present a 60-year-old lady presented with painless mass in the right calf for 2 years.

The mass was soft, non-tender, mobile from side to side. Ultrasound of the mass showed cystic lesion that contained multiple small cysts inside, and the diagnosis of hydatid disease was made before surgery.

During surgery and after opening the mass the lesion appeared to be hydatid cyst containing innumerable daughter cysts. Complete evacuation of the all the daughter cysts done. The patient discharged next day with no postoperative complications.

The patient received postoperative albendazole therapy for 2 months and follow up done 6 months after surgery with no complications.

**Conclusion:**

Hydatid disease should be thought when patients present with mass lesion in any part of the body especially in endemic areas. The most important step in the diagnosis is to exclude the presence of other cysts in other parts of the body.

## Highlights

• Hydatid disease is endemic in certain parts of the globe.
• High index of suspicion is required for the diagnosis of this disease specially in rare anatomical sites like the calf.
• Involvement of other organs must be excluded as the disease may affects many organs simultaneously.

## 1 Introduction

Hydatid disease is a zoonotic disease that is transmitted to the human by ingesting the eggs of the parasite *Echinococcus granulosus*, it commonly affects the liver and the lungs but every organ could be affected. Involvement of the musculoskeletal system is very rare comprising about 0.5–4% [[1], [2], [3]].

Hydatid cyst affecting the muscles present as soft tissue mass in the affected muscle, the mass may resemble a soft tissue tumor [2,4].

Preoperative diagnosis is very important and it is usually done radiologically by ultrasound, which may be the preferred diagnostic tool in some cases, or MRI examination which shows the characteristic radiological appearance of the cyst [5,6].

The radiological appearance of the disease varies from cystic lesion containing clear fluid, there may be rim calcifications of the edges, multi-cystic appearance, or calcified cyst [7,8].

The work in this article has been reported in line with the SCARE criteria [9].

### 1.1 Patient information

We present a 60-year-old lady presented to the surgical consultation room complaining from painless mass in the right calf for the last 2 years.

The mass accidentally discovered 2 years before presentation which was diagnosed that time clinically as lipoma, the patient didn’t receive any treatment at that time.

In the last few months the mass increased gradually in size and the patient was aware about this change in the size.

The patient has hypertension for 10 years controlled with antihypertensive drugs, the past surgical history of the patient included history of laparoscopic cholecystectomy before 10 years and hysterectomy for large uterine fibroid before 5 years. The patient didn’t receive anthelminthic medication before surgery.

### 1.2 Clinical findings

During examination there was a 10 cm by 7 cm soft mass in the posterior aspect of the right calf region. The mass was soft, non-tender, mobile from side to side.

### 1.3 Diagnostic assessment

Ultrasound of the mass showed cystic lesion that contained multiple small cysts inside, and the diagnosis of hydatid disease was made before surgery. Chest X-ray was taken before surgery and pulmonary involvement was excluded. Abdominal ultrasound was done and showed no involvement of the intra-abdominal organs.

### 1.4 Therapeutic intervention

**Fig. 1:**
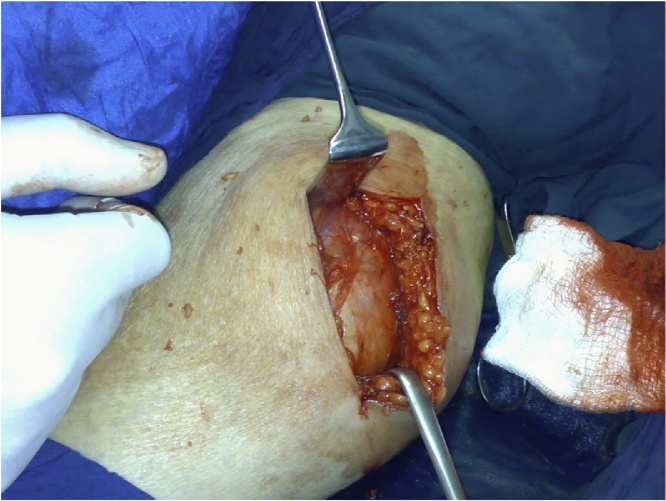
Intraoperative picture showing the cystic lesion in the calf before being opened.

**Fig. 2:**
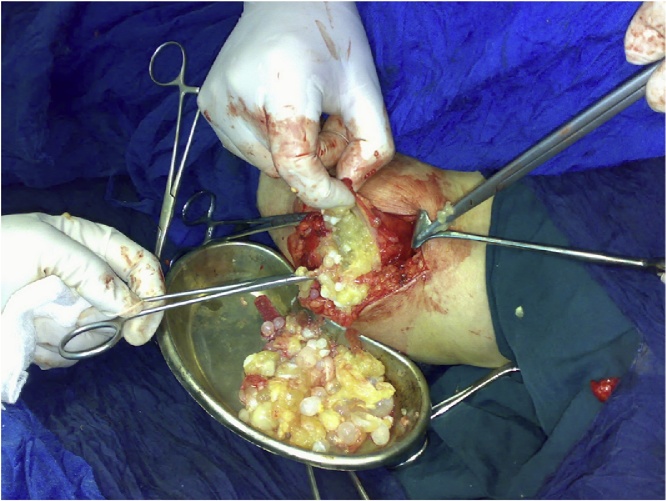
Intraoperative picture showing the mass containing innumerable daughter cysts.

During surgery cyst opened and was containing innumerable daughter cysts (Fig. 1, Fig. 2).

**Fig. 3:**
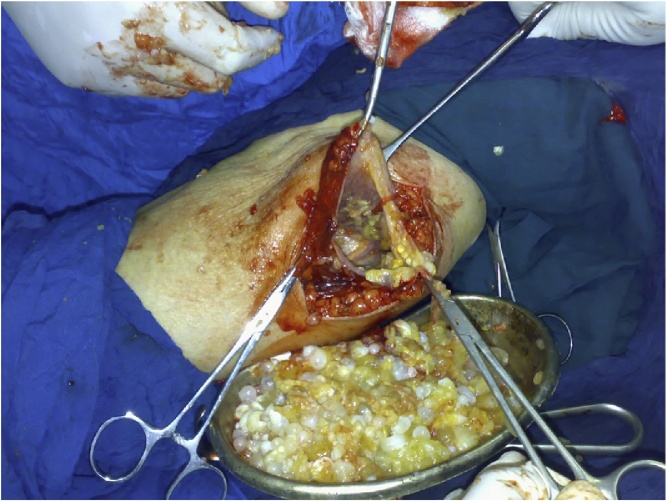
Intraoperative picture showing the cavity of the cyst after being evacuated from the contents.

Complete evacuation of the all the daughter cysts done, and the cavity of the cyst washed by chlorhexidine solution (Fig. 3).

### 1.5 Follow-up and outcomes

The patient discharged next day with no postoperative complications. The patient received postoperative albendazole therapy for 2 months. Follow up ultrasounds done on 2 occasions after surgery, 3 months apart with normal ultrasound examination. The patient advised for medical consultation if any kind of symptoms developed.

## 2 Discussion

Hydatid disease is endemic in certain parts of the world like certain parts of the middle east, some parts of India, south America, Australia, New Zealand [10].

The presentation of hydatid disease depends of the organ involved, Hydatid cyst of the musculoskeletal system presents as mass lesion, however it is very important to exclude other organ involvement particularly the lungs and the liver before any operative intervention as primary involvement of the musculoskeletal system is very rare [5].

Affection of the muscle is rare due to the high contents of lactic acid in the muscle which is not a suitable environment for parasite growth which needs high level of oxygen for its growth [11].

Clinical examination usually shows a painless soft tissue mass of the affected muscle, there may be elevated eosinophil count in the blood film and the serological test is positive in the majority of patients with active disease [11].

Many cases of hydatid cyst of the muscles have been reported worldwide. The treatment modalities depend on the anatomical site of the cyst and its relation to the major anatomical structures, the number, and the experience of the surgeon, this may include complete excision of the cyst without destroying the cyst wall. When the anatomical site of the cyst is difficult or in case of recurrent cyst, it can be managed using the PAIR method which is the abbreviation of the following, P: puncturing the cyst, A: aspiration of the fluid contents, I: injection of scolicidal agents such as hypertonic saline, chlorhexidine solution, povidone iodine solution, ethanol or formaldehyde solution in low concertation, the last 2 solution are not used in other locations like the liver because of the risk of biliary destruction an cirrhosis, and the R: re-aspiration of the fluid contents after the injection. In cases of recurrent cysts when complete excision is not possible, excision of the cyst alone may be the best option of treatment [[11], [12], [13], [14], [15], [16]].

Postoperative albendazole therapy was given for six weeks in the intend of decreasing recurrence. The anthelminthic medications are recommended by most of the authors after surgery in the intent of reducing the recurrence rates, the duration of medical therapy is variable but in most of the trials it ranges between 2–3 months. Long term follow up is required to exclude recurrence and to exclude other organ involvement [5,8].

## Conflicts of interest

The author has no conflicts of interest to declare.

## Funding

None.

## Ethical approval

Ethical approval has been exempted by my institution for reporting this case.

## Consent

Written informed consent was obtained from the patient for publication of this case report and accompanying images.

## Author contribution

Dr Ayad Ahmad Mohammed and Dr Sardar Hassan Arif. contributed to the concept of reporting the case and the patient data recording.

Dr Sardar Hassan Arif and Dr Ayad Ahmad Mohammed are the surgeons who performed the surgery.

Drafting the work, design, and revision done by Dr Ayad Ahmad Mohammed.

Dr Ayad Ahmad Mohammed took the consent from the patient for publishing the case.

Final approval of the work to be published was done by Dr Ayad Ahmad and Dr Sardar Hassan Arif.

## Registration of research studies

This work is case report and there is no need of registration.

## Guarantor

Dr Ayad Ahmad Mohammed is guarantor for the work.

## Patient perspective

The patient was worried about the possibility of other cysts in other parts of the body but after clinical and imaging evaluation, we excluded this possibility. The surgery done with no complications and the patients informed for regular medical follow up.

## Provenance and peer review

Not commissioned, externally peer-reviewed.
